# Procrustes Analysis for High-Dimensional Data

**DOI:** 10.1007/s11336-022-09859-5

**Published:** 2022-05-18

**Authors:** Angela Andreella, Livio Finos

**Affiliations:** 1 Department of Economics, CA’ Foscari University of Venice, San Giobbe - Cannaregio 873, Fondamenta San Giobbe, 30121 Venice, Italy; 2 Department of Developmental Psychology and Socialization, University of Padova, Via Venezia, 8, Padua, Italy

**Keywords:** functional alignment, functional magnetic resonance imaging, high-dimensional data, Procrustes analysis, Von Mises–Fisher distribution

## Abstract

The Procrustes-based perturbation model (Goodall in J R Stat Soc Ser B Methodol 53(2):285–321, 1991) allows minimization of the Frobenius distance between matrices by similarity transformation. However, it suffers from non-identifiability, critical interpretation of the transformed matrices, and inapplicability in high-dimensional data. We provide an extension of the perturbation model focused on the high-dimensional data framework, called the ProMises (Procrustes von Mises–Fisher) model. The ill-posed and interpretability problems are solved by imposing a proper prior distribution for the orthogonal matrix parameter (i.e., the von Mises–Fisher distribution) which is a conjugate prior, resulting in a fast estimation process. Furthermore, we present the Efficient ProMises model for the high-dimensional framework, useful in neuroimaging, where the problem has much more than three dimensions. We found a great improvement in functional magnetic resonance imaging connectivity analysis because the ProMises model permits incorporation of topological brain information in the alignment’s estimation process.

**Supplementary Information:**

The online version contains supplementary material available at 10.1007/s11336-022-09859-5.

The Procrustes problem is aimed at matching matrices using similarity transformations by minimizing their Frobenius distance. It allows comparison of matrices with dimensions defined in an arbitrary coordinate system. This method raised the interest of applied researchers hence highlighting its potentiality through a plethora of applications in several fields, such as ecology (Saito et al., 2015), biology (Rohlf & Slice, 1990), analytical chemometrics (Andrade et al., 2004), and psychometrics (Green, 1952; McCrae et al., 1996).

The interest of a large audience from applied fields stimulates, in parallel, the growth of a vast body of literature. Despite this, essentially all applications comprise spatial coordinates (i.e., two- or three-dimensional). Haxby et al. (2011) first introduced the use of this approach into a different context: align functional Magnetic Resonance Images (fMRI). The coordinates are hence substituted by voxels (i.e., three-dimensional pixels), and the problem becomes inherently high-dimensional. The approach rapidly grew in popularity in the neuroimaging community because of its effectiveness. However, the proposed solution is naive; the extension from the spatial context to a more general and high-dimensional one is a theoretical challenge that needs adequate attention.

The most serious concern is the results’ interpretability. In most cases, Procrustes methods turn into an ill-posed problem. It is a barely noticeable problem with spatial coordinates because the solution is unique up to rotations; hence, the user has the freedom to choose the point of view that provides the nicest picture. When the dimensions do not have a spatial meaning, any rotation completely changes the interpretation of the results.

To tackle this problem, we revise the perturbation model (Goodal, 1991), which rephrases the Procrustes problem as a statistical model. The matrices are defined as a random perturbation of a reference matrix plus an error term. The perturbation is expressed by rotation, scaling, and translation, and the matrix normal distribution (Gupta & Nagar, 2018) is assumed for the error terms. Like Green and Mardia (2013) and Mardia et al. (2006), we assume that the orthogonal matrix parameter follows the von Mises–Fisher distribution. We prove that the proposed prior distribution is conjugate, making the estimation process quite fast. Indeed, the maximum a posterior estimate of the orthogonal matrix parameters results in a minor modification of the original solution because the prior information enters into the pairwise cross-product of the matrices to be aligned. The prior distribution plays the role of regularizing term, resolving the non-identifiability of the orthogonal matrix parameter. In the application to fMRI data in Sect. 4, we further show that specification of a prior distribution permits the integration of functional and topological aspects, which largely improves the results’ interpretability. We then propose a comprehensive approach to the Procrustes problem: the ProMises (Procrustes von Mises–Fisher) model.

The second problem raised by the extension to the high-dimensional framework is computational. The estimation algorithm of a Procrustes-based method involves a series of singular value decompositions of $m\times m$ matrices (*m* dimensions). In a typical fMRI data set, the subjects (i.e., the matrices to be aligned) have a few hundred (observations/rows) *n* and hundreds of thousands of voxels (dimensions/columns) *m*. We prove that the minimization problem can be solved by a series of singular value decompositions of $n\times n$ matrices, reducing the computation burden and making the ProMises model applicable to matrices with virtually any number of columns. We denote this approach as the Efficient ProMises model.

We emphasize here that the problem of aligning fMRI data is not three-dimensional as it could appear at first glance, although it is high-dimensional: Each voxel is one dimension of the problem. Nevertheless, in such conditions, three critical issues arise: The first is that the Procrustes method combines any voxel inside the brain without distinguishing between adjacent and distant anatomical locations. This can be questionable because the voxels have a spatial organization, and, despite the inter-subject variability, we expect some degree of spatial similarity between subjects in their functional organization. Therefore, we want the subjects’ voxels of a given location to be more likely to contribute to the construction of voxels with the same location in the common space. The second issue revolves around the non-identifiability of orthogonal transformations ${\varvec{R}}_i$, where *i* indexes the subjects. The Procrustes method does not return a unique solution of the maximum likelihood estimate for ${\varvec{R}}_i$: Given a solution (i.e., a three-dimensional image), any linear combination that mixes the voxels’ values is an equivalent solution to the problem. The solutions are equivalent only from a mathematical point of view because the practical consequence is the loss of results’ topological interpretability. The third issue is the computational load: applying the Procrustes-based alignment to the whole brain implies the decomposition of many square matrices of dimensions roughly equal to 200.000 (i.e., the number of voxels.)

The Efficient ProMises model resolves all these three issues. The use of a properly chosen prior shrinks the estimate to the anatomical solution (i.e., no rotation), hence making the solution unique and interpretable from an anatomical point of view. Finally, as mentioned before, the Efficient implementation permits performing functional alignment on high-dimensional data such as fMRI data.

The paper is organized as follows. Section 1 introduces the perturbation model (Goodall, 1991), stressing its critical issues. Section 2 illustrates the ProMises model and its challenges: identifiability, interpretability, and flexibility. Section 3 defines the Efficient version of the ProMises model, which permits application of the functional alignment to high-dimensional data. Finally, the presented model is evaluated by analyzing task-related fMRI data in Sect. 4. The entire code used is available in https://github.com/angeella/ProMisesModel using the programming language Python (Van Rossum and Drake Jr, 1995) and in https://github.com/angeella/alignProMises using the R (R Core Team, 2018) package alignProMises. We report the proofs of the main lemmas here, whereas the remaining proofs are included in the supplementary material.

## Perturbation Model

### Background

Let $\{{\varvec{X}}_{i} \in \mathrm{I\!R}^{n \times m} \}_{i = 1,\dots ,N}$ be a set of matrices to be aligned. The Procrustes-based method uses similarity transformations to match each matrix to the target one as closely as possible, according to the Frobenius distance.

Each matrix ${\varvec{X}}_i$ could be then assumed to be a similarity transformation of a shared matrix ${\varvec{M}} \in \mathrm{I\!R}^{n \times m}$, which contains the common reference space’s coordinates, plus a random error matrix ${\varvec{E}}_i \in \mathrm{I\!R}^{n \times m}$. The perturbation model proposed by Goodall (1991) is then reported.

#### Definition 1

(*Goodall*, 1991) Let $\{{\varvec{X}}_{i} \in \mathrm{I\!R}^{n \times m} \}_{i = 1,\dots ,N}$ be a set of matrices to be aligned and ${\mathcal {O}}(m)$ the orthogonal group in dimension *m*. The perturbation model is defined as

$$\begin{aligned} {\varvec{X}}_i= \alpha _i ({\varvec{M}} + {\varvec{E}}_i){\varvec{R}}_i^\top + {\mathbf {1}}_n^\top {\varvec{t}}_i \quad \quad \text {subject to }\quad {\varvec{R}}_i \in {\mathcal {O}}(m), \end{aligned}$$

where ${\varvec{E}}_i \sim \mathcal{MN}\mathcal{}_{n,m}(0,\varvec{\Sigma }_n,\varvec{\Sigma }_m)$ —i.e., the matrix normal distribution with $\varvec{\Sigma }_n \in \mathrm{I\!R}^{n \times n}$ and $\varvec{\Sigma }_m \in \mathrm{I\!R}^{m \times m}$ scale parameters— ${\varvec{M}} \in \mathrm{I\!R}^{n \times m}$ is the shared matrix, $\alpha _i \in \mathrm{I\!R}^{+}$ is the isotropic scaling, ${\varvec{t}}_i \in \mathrm{I\!R}^{1 \times m}$ defines the translation vector, and ${\mathbf {1}}_n \in \mathrm{I\!R}^{1 \times n}$ is a vector of ones.

To simplify the problem, the column-centered ${\varvec{X}}_i$ are considered. The distribution is

$$\begin{aligned} \begin{aligned} \text{ vec }({{C}}_n{{X}}_i| {{R}}_i, \alpha _i, {{M}}, {\Sigma }_m, {\Sigma }_n) \sim {\mathcal {N}}_{n m}(\text{ vec }(\alpha _i {{C}}_n{{M}} {{R}}_i^\top ), {{R}}_i {\Sigma }_m {{R}}_i^\top \otimes \alpha _i^2 {{C}}_n {\Sigma }_n {{C}}_n^\top ), \end{aligned}\end{aligned}$$

where ${\varvec{C}}_n = {\varvec{I}}_n - \frac{1}{n} {\varvec{J}}_n$, ${\varvec{I}}_n \in \mathrm{I\!R}^{n \times n}$ is the identity matrix, ${\varvec{J}}_n$ is a $n \times n$ matrix of ones, and $\text {vec}({\varvec{A}})$ the vectorization of the matrix ${\varvec{A}}$. Let the singular value decomposition of ${\varvec{C}}_n = \varvec{\Gamma } \varvec{\Delta } \varvec{\Gamma }^\top$, where $\varvec{\Gamma } \in \mathrm{I\!R}^{n \times (n-1)}$, then:

$$\begin{aligned} \begin{aligned} \text{ vec }({\Gamma }^\top {{C}}_n{{X}}_i| {{R}}_i, \alpha _i, {{M}}, {\Sigma }_m, {\Sigma }_n) \sim&{\mathcal {N}}_{(n-1) m}(\text{ vec }(\alpha _i {\Gamma }^\top {{C}}_n{{M}} {{R}}_i^\top ), \\&\quad {{R}}_i {\Sigma }_m {{R}}_i^\top \otimes \alpha _i^2 {\Gamma }^\top {{C}}_n {\Sigma }_n {{C}}_n^\top {\Gamma }). \end{aligned}\end{aligned}$$

The $\varvec{\Gamma }^\top$ transformation leads to independence between the rows of ${\varvec{E}}_i$ without affecting the estimation of ${\varvec{R}}_i$. $\varvec{\Gamma }^\top {\varvec{C}}_n {\varvec{X}}_i$ and $\varvec{\Gamma }^\top {\varvec{C}}_n {\varvec{M}}$ have now $n-1$ rows; however, we can simply re-project them on $\mathrm{I\!R}^{n \times m}$ using $\varvec{\Gamma }$. Because we can always write $\tilde{{\varvec{X}}}_i = \varvec{\Gamma } \varvec{\Gamma }^\top {\varvec{C}}_n {\varvec{X}}_i$, $\tilde{{\varvec{M}}} = \varvec{\Gamma } \varvec{\Gamma }^\top {\varvec{C}}_n {\varvec{M}}$, and $\tilde{\varvec{\Sigma }}_n = \varvec{\Gamma }\varvec{\Gamma }^\top {\varvec{C}}_n \varvec{\Sigma }_n {\varvec{C}}_n^\top \varvec{\Gamma }\varvec{\Gamma }^\top$ without loss of generality, we can re-write Definition 1 as follows:

#### Definition 2

1 $$\begin{aligned} {\varvec{X}}_i= \alpha _i ({\varvec{M}} + {\varvec{E}}_i){\varvec{R}}_i^\top \quad \quad \text {subject to }\quad {\varvec{R}}_i \in {\mathcal {O}}(m) , \end{aligned}$$

where ${\varvec{E}}_i \sim \mathcal{MN}\mathcal{}_{n m}(0,\varvec{\Sigma }_n,\varvec{\Sigma }_m)$. This way, we obtain the following:

2 $$\begin{aligned} \text {vec}({\varvec{X}}_i| {\varvec{R}}_i, \alpha _i, {\varvec{M}}, \varvec{\Sigma }_m, \varvec{\Sigma }_n) \sim {\mathcal {N}}_{n m}(\text {vec}(\alpha _i {\varvec{M}} {\varvec{R}}_i^\top ), {{R}}_i {\Sigma }_m {{R}}_i^\top \otimes \alpha _i^2 {\Sigma }_n). \end{aligned}$$

The following notation is also adopted: $||\cdot ||$ to indicate the Frobenius norm and $<\cdot ,\cdot>$ for the Frobenius inner product (Golub & van Loan, 2013).

The main objective of this work is comparing the shapes ${\varvec{X}}_i$ instead of the form’s analysis of the matrices. For that, the parameters of interest are ${\varvec{R}}_i$ and $\alpha _i$, whereas ${\varvec{M}}$, $\varvec{\Sigma }_n$, and $\varvec{\Sigma }_m$ are considered as nuisance parameters for each $i = 1, \dots , N$. The estimation of the unknown parameters changes if these nuisance parameters are known. Section 1.2 initially presents the estimates under this assumption and then provides the more realistic case of unknown nuisance parameters.

### Estimation of the Perturbation Model

We formalize some results from Theobald and Wuttke (2006) in the case of known nuisance parameters ${\varvec{M}}$, $\varvec{\Sigma }_m$, and $\varvec{\Sigma }_n$, with $\varvec{\Sigma }_n$ and $\varvec{\Sigma }_m$ positive definite matrices by the following theorem:

#### Theorem 1

(Theobald & Wuttke 2006) Consider the perturbation model described in Definition 2, and the singular value decomposition ${\varvec{X}}_i^\top \varvec{\Sigma }_n^{-1} {\varvec{M}} \varvec{\Sigma }_m^{-1}= {\varvec{U}}_i {\varvec{D}}_i {\varvec{V}}_i^\top$. The maximum likelihood estimators equal $\hat{{\varvec{R}}}_i = {\varvec{U}}_i {\varvec{V}}_i^\top$, and $\hat{\alpha _i}_{\varvec{{\hat{R}}}_i} = ||\varvec{\Sigma }_m^{-1/2} \hat{{\varvec{R}}}_i^\top {\varvec{X}}_i^\top \varvec{\Sigma _n}^{-1/2}||^2/tr{({\varvec{D}}_i)}$.

Now consider *N* independent observations ${\varvec{X}}_1, \dots , {\varvec{X}}_N$. The joint log-likelihood is simply the sum of *N* log-likelihoods.

In the case of unknown nuisance parameters ${\varvec{M}}$, $\varvec{\Sigma }_m$, and $\varvec{\Sigma }_n$, the joint likelihood cannot be written as product of separated likelihoods, one for each ${\varvec{X}}_i$, because each of the unknown parameters is a function of the others. The solution must be found by an iterative algorithm. In particular, the two covariance matrices $\varvec{\Sigma }_m$ and $\varvec{\Sigma }_n$ can be estimated by a two-stage algorithm defined in Dutilleul (1999), where $\varvec{{\hat{\Sigma }}}_n = \{\sum _{i=1}^{N}({\varvec{X}}_i - \varvec{{\hat{M}}}) \varvec{{\hat{\Sigma }}}_m^{-1} ({\varvec{X}}_i - \varvec{{\hat{M}}})^\top \}/Nm$ and $\varvec{{\hat{\Sigma }}}_m = \{\sum _{i=1}^{N}({\varvec{X}}_i - \varvec{{\hat{M}}})^\top \varvec{{\hat{\Sigma }}}_n^{-1} ({\varvec{X}}_i - \varvec{{\hat{M}}})\}/Nn$ are maximum likelihood estimators.

The necessary and sufficient condition for the existence of $\varvec{{\hat{\Sigma }}}_n$ and $\varvec{{\hat{\Sigma }}}_m$ is $N \ge \frac{m}{n} + 1$, assuming $\varvec{\Sigma }_m$ and $\varvec{\Sigma }_n$ are positive definite matrices. In real applications, this assumption could be problematic. For example, in fMRI data analysis, *m* roughly equals 200, 000, and n approximately equals 200; therefore, the researcher would have to analyzing at least 1, 001 subjects, which is virtually impossible because fMRI is costly.

Various solutions can be found in the literature: Theobald and Wuttke (2006) proposed a regularization for the covariance matrix, whereas Lele (1993) estimated $\varvec{\Sigma }_n$ using the distribution of ${\varvec{X}}_i {\varvec{X}}_i^\top$. In this work, we maintain a general formulation of the estimator for $\varvec{{{\hat{\Sigma }}}}_m = g(\varvec{{\hat{\Sigma }}}_n, \varvec{{\hat{M}}}, {\varvec{X}}_i)$ and $\varvec{{{\hat{\Sigma }}}}_n = g(\varvec{{\hat{\Sigma }}}_m, \varvec{{\hat{M}}}, {\varvec{X}}_i)$, because we aim to find a proper estimate of ${\varvec{R}}_i$ rather than $\varvec{\Sigma }_n$ and $\varvec{\Sigma }_m$. The shared matrix ${\varvec{M}}$ is estimated by the element-wise arithmetic mean of $\{\hat{{\varvec{X}}}_i\}_{i = 1, \dots , N}$, where $\hat{{\varvec{X}}}_i = {\hat{\alpha }}_{\hat{{\varvec{R}}}_i}^{-1} {\varvec{X}}_i \hat{{\varvec{R}}}_i$. We then modified the iterative algorithm of Gower (1975) (i.e., generalized Procrustes analysis), to estimate ${\varvec{R}}_i$.
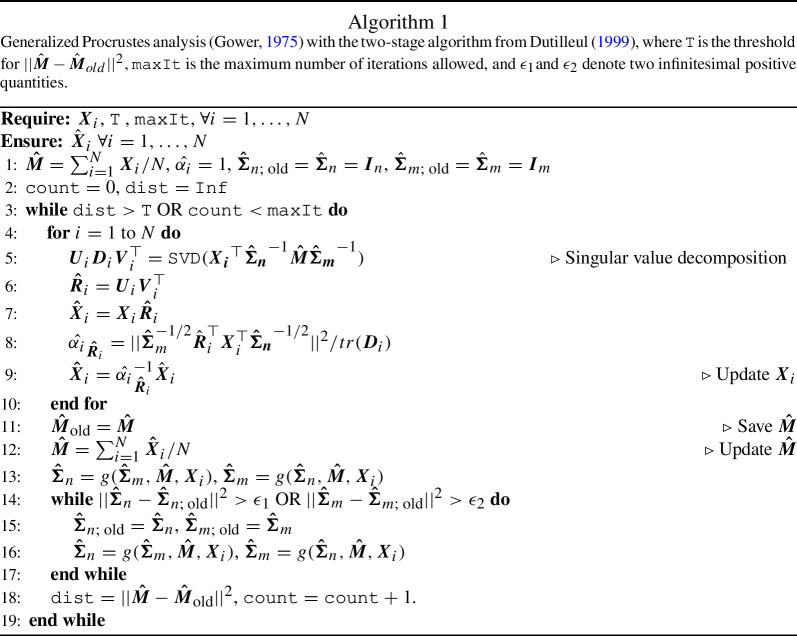


Groisser (2005) proved the convergence of the generalized Procrustes analysis algorithm. However, it leads to non-identifiable estimators of ${\varvec{R}}_i$, $i = 1, \dots , N$, proved by the following lemma:

#### Lemma 1

Let $\{\varvec{{\hat{R}}}_i\}_{i=1,\dots ,N}$ be the maximum likelihood solutions for $\{{\varvec{R}}_i\}_{i=1,\dots ,N}$ with ${\varvec{M}}$, $\varvec{\Sigma }_n$, and $\varvec{\Sigma }_m$ as unknown parameters. If ${\varvec{Z}} \in {\mathcal {O}}(m)$, then $\{\varvec{{\hat{R}}}_i {\varvec{Z}}\}_{i = 1, \dots , N}$ are still valid maximum likelihood solutions for $\{\varvec{{\hat{R}}}_i\}_{i = 1, \dots , N}$.

#### Proof

Consider $${\varvec{Z}} \in {\mathcal {O}}(m)$$, and

$$\begin{aligned} \dfrac{1}{\alpha _i} {\varvec{X}}_i {\varvec{R}}_i {\varvec{Z}} - {\varvec{M}}{\varvec{Z}} = {\varvec{E}}_i {\varvec{Z}} \sim \mathcal{MN}\mathcal{}(0, \varvec{\Sigma }_n, {\varvec{Z}}^\top \varvec{\Sigma }_m {\varvec{Z}}). \end{aligned}$$

Consider the proof of Theorem 1 placed in the supplementary material, we have

3 $$\begin{aligned} \max _{{\varvec{R}}_i \in {\mathcal {O}}(m)} \sum _{i = 1}^{N} <{\varvec{R}}_i, {\varvec{X}}_i^\top \varvec{\Sigma }_n^{-1} {\varvec{M}} \varvec{\Sigma }_m^{-1}> = \max _{{\varvec{R}}_i \in {\mathcal {O}}(m)} \sum _{i =1}^{N} tr({\varvec{Z}}^\top {\varvec{R}}_i^\top {\varvec{X}}_i^\top \varvec{\Sigma }_n^{-1} {\varvec{M}} {\varvec{Z}} {\varvec{Z}}^\top \varvec{\Sigma }_m^{-1} {\varvec{Z}}). \end{aligned}$$

Since ${\varvec{Z}}^\top {\varvec{Z}} = {\varvec{Z}} {\varvec{Z}}^\top = {\varvec{I}}_m$, the solutions $\{\hat{{\varvec{R}}}_i {\varvec{Z}}\}_{i = 1, \dots , N}$ are still valid solutions for the maximization (3). $\square$

To sum up, in the more realistic case, when we must estimate the nuisance parameters, the Procrustes solutions are infinite in general, in both in the high-dimensional case and the low-dimensional by Lemma 1. We emphasize here that, to resolve the non-identifiability of ${\varvec{R}}_i$, the proposed ProMises model imposes a prior distribution for the parameter ${\varvec{R}}_i$.

## ProMises Model

### Background

In the previous section, we justified how the perturbation model could be problematic because Lemma 1 proves the non-identifiability of the parameter ${\varvec{R}}_i$ in the realistic case of unknown nuisance parameters. This scenario returns to be critical in several applications, where the *m* columns of the matrix ${\varvec{X}}_i$ do not express the three-dimensional spatial coordinates, such as in the fMRI data framework illustrated in Sect. 4. In the high-dimensional case, the final orientations of the aligned data can be relevant for interpretation purposes.

For that, we propose its Bayesian approach: the ProMises model. We stress here that a proper prior distribution leads to a closed-form and interpretable, unique point estimate of ${\varvec{R}}_i$. The specification of the prior parameters is essential, especially in our high-dimensional context.

### Interpretation of the Prior Parameters

Because ${\varvec{R}}_i \in {\mathcal {O}}(m)$, a proper prior distribution must take values in the Stiefel manifold $V_{m}(\mathrm{I\!R}^m)$. The matrix von Mises–Fisher distribution is a non-uniform distribution on $V_{m}(\mathrm{I\!R}^m)$, which describes a rigid configuration of *m* distinct directions with fixed angles. It was proposed by Downs (1972) and investigated by many authors (e.g., Chikuse, 1979; Jupp & Mardia 2003).

We report below the formal definition of the von Mises–Fisher distribution.

#### Definition 3

(*Downs*, 1972) The von Mises–Fisher distribution for ${\varvec{R}}_i \in {\mathcal {O}}(m)$ is

4 $$\begin{aligned} f({\varvec{R}}_i) = C({\varvec{F}}, k ) \exp \big \{tr(k {\varvec{F}}^\top {\varvec{R}}_i)\big \} , \end{aligned}$$

where $C({\varvec{F}}, k)$ is a normalizing constant, ${\varvec{F}} \in \mathrm{I\!R}^{m \times m}$ is the location matrix parameter, and $k \in \mathrm{I\!R}^{+}$ is the concentration parameter.

The parameter *k* defined in (4) balances the amount of concentration of the distribution around ${\varvec{F}}$. If $k \rightarrow 0$, the prior distribution is near a uniform distribution (i.e., unconstrained). If $k \rightarrow +\infty$, the prior distribution tends toward a Dirac distribution (i.e., maximum constraint).

A proper specification of the prior distribution leads to improved estimation of ${\varvec{R}}_i$. Therefore, the core of the ProMises model is the specification of ${\varvec{F}}$ defined in (4). Consider the polar decomposition and singular value decomposition of ${\varvec{F}}= {\varvec{P}} {\varvec{K}} = {\varvec{L}} \varvec{\Sigma }{\varvec{B}}^\top = {\varvec{L}} {\varvec{B}}^\top {\varvec{B}} \varvec{\Sigma } {\varvec{B}}^\top$, where ${\varvec{P}}, {\varvec{L}}, {\varvec{B}} \in {\mathcal {O}}(m)$, and ${\varvec{K}} \in \mathrm{I\!R}^{m \times m}$ are symmetric positive semi-definite matrices, and $\varvec{\Sigma } \in \mathrm{I\!R}^{m \times m}$ diagonal matrix with non-negative real numbers on the diagonal. The mode of the density defined in (4) equals ${\varvec{P}}$ (Jupp & Mardia, 1979), so the most plausible rotation matrix depends on the orientation characteristic of ${\varvec{F}}$. Merging the two decompositions, ${\varvec{P}} = {\varvec{L}} {\varvec{B}}^\top$ describes the orientation part of ${\varvec{F}}$, and ${\varvec{K}} = {\varvec{B}} \varvec{\Sigma } {\varvec{B}}^\top$ defines the concentration part. The mode is specified by the product of the left and right singular vectors of ${\varvec{F}}$. These decompositions are useful to understand when the density (4) is uni-modal. If ${\varvec{F}}$ has full rank, $\varvec{\Sigma }$ does too, the polar decomposition is unique, and thus the mode of the density (i.e., ${\varvec{P}}$ is the global maximum). Let ${\varvec{F}}$ be a full rank matrix, then the maximum equals $\max _{{\varvec{R}}_i \in {\mathcal {O}}(m)} \,\, tr({\varvec{F}} {\varvec{R}}_i^\top ) = tr\{{\varvec{L}} {\varvec{B}}^\top {\varvec{B}} \varvec{\Sigma } {\varvec{B}}^\top ({\varvec{L}} {\varvec{B}}^\top )^\top \} = tr(\varvec{\Sigma })$.

To sum up, the prior specification allows us to include a priori information about the optimal orientation in the perturbation model. We anticipate here the result of Lemma 3: If ${\varvec{F}}$ is defined as a full rank matrix, the maximum a posteriori solution $\varvec{{\hat{R}}^{'}}_i$ will be unique with the orientation structure of ${\varvec{F}}$.

Two simple examples of ${\varvec{F}}$ are delineated below.

#### Example 1

The most simple definition of ${\varvec{F}}$ is ${\varvec{I}}_m$ (Lee, 2018). The eigenvalues are all 1, and ${\varvec{L}}$ and ${\varvec{B}}$ are equal to ${\varvec{e}}_1,\dots , {\varvec{e}}_m$, where ${\varvec{e}}_i$ is the standard basis forming an orthonormal basis of $\mathrm{I\!R}^{m}$. The prior distribution shrinks the possible solutions for ${\varvec{R}}_i$ toward orthogonal matrices that consider only the combination of variables with the same location.

Alternatively, considering the fMRI scenario, the hyperparameter ${\varvec{F}}$ can be defined as an Euclidean similarity matrix using the 3*D* anatomical coordinates *x*, *y*, and *z* of each voxel:

$$\begin{aligned} {\varvec{F}} = \Big [\exp \Big \{- \sqrt{(x_i - x_j)^2 + (y_i - y_j)^2 + (z_i - z_j)^2}\Big \}\Big ], \end{aligned}$$

where $i,j = 1,\dots m$. In this way, ${\varvec{F}}$ is a symmetric matrix with ones in the diagonal, which means that voxels with the same spatial location are combined with weights equalling 1, and the weights decrease as the voxels to be combined are more spatially distant.

#### Example 2

Consider *N* matrices, one for each plant, describing the three-dimensional spatial trajectories of a climbing plant, Pisum sativum, having wooden support as a stimulus (Guerra et al., 2019) across time. The spatiotemporal trajectories of the plants are analyzed until they come to grasp the stick. The aim is to functionally align the three time series, one for each coordinate (*x*, *y*, *z*); then, we have $m=3$. In this case, we could suppose that the rotation along the *z* axis is not of interest because the functional misalignment between plants can be along the *x*-axis and *y*-axis with the *z*-axis being the one that reflects the growth of the plants and the *x*-axis and *y*-axis describing the elliptical movement (circumnutation) of the plants (Guerra et al., 2019). Therefore, the ${\varvec{F}}$ location matrix parameter can be described as follows:

5 $$\begin{aligned} {\varvec{F}}=\begin{bmatrix} 0.5 &{}\quad 0.5 &{}\quad 0 \\ 0.5 &{}\quad 0.5 &{}\quad 0 \\ 0 &{}\quad 0 &{}\quad 1 \\ \end{bmatrix}. \end{aligned}$$

The axes *x* and *y* have the same probability of entering in the calculation of the first two dimensions of the final common space, whereas the *z* axis is not considered.

**Fig. 1:**
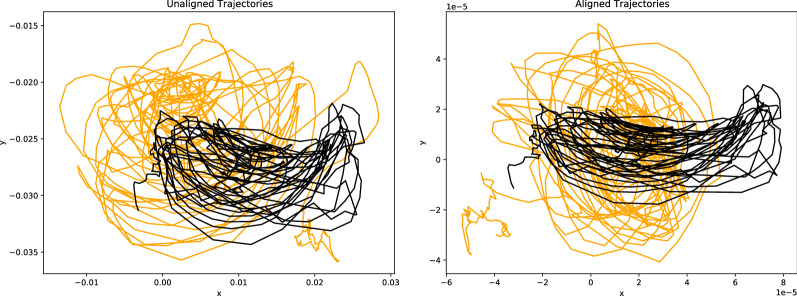
Left panel: Unaligned spatial trajectories of the tendrils of two plants. Right panel: Aligned spatial trajectories of the tendrils of two plants.

We use the kinematic plant data from Guerra et al. (2019), consisting of five matrices/plants. Figure 1 shows the elliptical movement expressed by the axes *x* and *y*, in the case of unaligned and aligned plant trajectories. The rotation transformations are estimated by the ProMises model with ${\varvec{F}}$ expressed as (5). We do not go into detail about the meaning of the results because we have introduced this example to explain the usefulness of ${\varvec{F}}$. However, we can note how the ProMises model aligns the final coordinates of the tendrils (i.e., when the plant touches the wooden support).

### Von Mises–Fisher Conjugate Prior

The von Mises–Fisher distribution (4) was proved by Khatri and Mardia (1977) to be a member of the standard exponential family (Barndorff–Nielsen, 2014). Green and Mardia (2006) mentioned that the von Mises–Fisher distribution is a conjugate prior for the matrix normal distribution, which we formally prove in the following lemma under the perturbation model’s assumptions.

#### Lemma 2

Consider the perturbation model of Definition 2, with ${\varvec{R}}_i$ distributed according to (4), then the posterior distribution $f({\varvec{R}}_i| k, {\varvec{F}}, {\varvec{X}}_i)$ is a conjugate distribution to the von Mises–Fisher prior distribution with location posterior parameter equalling the following:

6 $$\begin{aligned} {\varvec{F}}^\star =\varvec{X_i}^\top \varvec{\Sigma }_n^{-1} {\varvec{M}} \varvec{\Sigma }_m^{-1} + k {\varvec{F}}. \end{aligned}$$

The posterior location parameter is the sum of ${\varvec{X}}_i^\top \varvec{\Sigma }_n^{-1} {\varvec{M}} \varvec{\Sigma }_m^{-1}$ and the prior location parameter ${\varvec{F}}$ multiplied by *k*. Consider the singular value decomposition of ${\varvec{X}}_i^\top \varvec{\Sigma }_n^{-1} {\varvec{M}} \varvec{\Sigma }_m^{-1}$:

7 $$\begin{aligned} {\varvec{X}}_i^\top \varvec{\Sigma }_n^{-1} {\varvec{M}} \varvec{\Sigma }_m^{-1} = {\varvec{U}}_i {\varvec{D}}_i {\varvec{V}}_i^\top = {\varvec{U}}_i {\varvec{V}}_i^\top {\varvec{V}}_i {\varvec{D}}_i {\varvec{V}}_i^\top . \end{aligned}$$

The right part of (7) ${\varvec{V}}_i {\varvec{D}}_i {\varvec{V}}_i^\top$ is the elliptical part of ${\varvec{X}}_i^\top \varvec{\Sigma }_n^{-1} {\varvec{M}} \varvec{\Sigma }_m^{-1}$, which is a measure of variation relative to the decomposition ${\varvec{U}}_i {\varvec{V}}_i^\top$ (i.e., the maximum likelihood estimator of ${\varvec{R}}_i$). Focus on the right part of (6), ${\varvec{F}}={\varvec{P}} {\varvec{K}}$, which is the polar decomposition of ${\varvec{F}}$, where ${\varvec{P}}$ is the mode of the von Mises–Fisher distribution, and ${\varvec{K}}$ is its measure of variation. Therefore, ${\varvec{F}}^\star$ is expressed as a combination of the maximum likelihood estimate $\varvec{{\hat{R}}}_i = {\varvec{U}}_i {\varvec{V}}_i^\top$ and the prior mode ${\varvec{P}}$, multiplied by corresponding measures of variation.

Thanks to the conjugacy, the estimation process remains simple; with a small modification we keep the previous algorithm without increasing the computational burden.

### Estimation of the ProMises Model

This section delineates the estimation process for ${\varvec{R}}_i$ using the ProMises method. First of all, $f(\varvec{X_i} | \alpha _i, \varvec{R_i})$ depends only on the product $\alpha _i \varvec{R_i}$, we thus refer to the distribution $f(\varvec{X_i} | \alpha _i \varvec{R_i})$ instead of $f(\varvec{X_i} | \alpha _i, \varvec{R_i})$ defined in (2). The following density is then considered as prior distribution for the product $\alpha _i \varvec{R_i}$:

8 $$\begin{aligned} f(\alpha _i \varvec{R_i}) \sim \exp \Big \{\dfrac{k}{\alpha _i}tr({\varvec{F}}^\top {\varvec{R}}_i)\Big \}\alpha _i^{-1}. \end{aligned}$$

The following theorem delineates the estimation of ${\varvec{R}}_i$ with known nuisance parameters:

#### Theorem 2

The ProMises model is defined as the perturbation model specified in Definition 2 imposing the prior distribution (8) for $\alpha _i {\varvec{R}}_i$. Let the singular value decomposition of ${\varvec{X}}_i^\top \varvec{\Sigma }_n^{-1} {\varvec{M}} \varvec{\Sigma }_m^{-1} + k {\varvec{F}}$ be ${\varvec{U}}_i {\varvec{D}}_i {\varvec{V}}_i^\top$. Then, the maximum a posteriori estimators equal $\varvec{{\hat{R}}^{'}}_i = {\varvec{U}}_i {\varvec{V}}_i^\top$ and $\hat{\alpha _i}_{\varvec{{\hat{R}}^{'}}_i}^{'}=||\varvec{\Sigma }_m^{-1/2} \varvec{{\hat{R}}^{'\top }}_i {\varvec{X}}_i^\top \varvec{\Sigma }_n^{-1/2}||^2/tr({\varvec{D}}_i)$.

The prior information about ${\varvec{R}}_i$’s structure is directly entered in the singular value decomposition step; the maximum a posteriori estimator turns out to be a slight modification of the solution given in Theorem 1. We decompose ${\varvec{X}}_i^\top \varvec{\Sigma }_n^{-1} {\varvec{M}} \varvec{\Sigma }_m^{-1} + k {\varvec{F}}$ instead of ${\varvec{X}}_i^\top \varvec{\Sigma }_n^{-1} {\varvec{M}} \varvec{\Sigma }_m^{-1}$.

Let $\{{\varvec{X}}_{i} \in \mathrm{I\!R}^{n \times m} \}_{i = 1,\dots ,N}$ be a set of independent matrices. Then, the joint posterior distribution is simply the product of the single posterior distribution.

If ${\varvec{M}}$, $\varvec{\Sigma }_n$, and $\varvec{\Sigma }_m$ are unknown, the maximization problem has no closed-form solution, like in Sect. 1.2. Because we proved that the prior specification modifies only the singular value decomposition step of Theorem 1, we then modify the Line 5 of Algorithm 1, as follows:
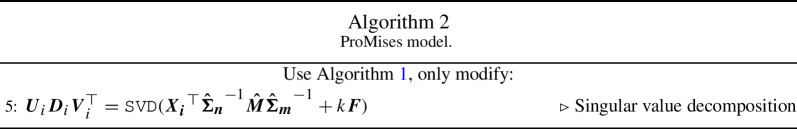


### On the Choice of the Parameter of the von Mises–Fisher Distribution

We choose the von Mises–Fisher distribution as prior distribution for ${\varvec{R}}_i$ for its useful and practical properties. First, as shown, it is a conjugate prior distribution, leading to a direct calculation and interpretation of $\varvec{{\hat{R}}^{'}}_i$. Second, it expresses the orthogonality constraint imposed by the Procrustes problem. Finally, the definition of ${\varvec{F}}$ does not require strong assumptions (Downs, 1972); nevertheless, if we specify it as a full-rank matrix, we guarantee solution’s uniqueness. This permits formulation of the below lemma.

#### Lemma 3

If ${\varvec{F}}$ has full rank, the maximum a posteriori estimates for ${\varvec{R}}_i$ given by Theorem 2 are unique.

#### Proof

Consider the proof of Lemma 1. Multiplying by ${\varvec{Z}}$ leads to the following maximization:

$$\begin{aligned} \sum _{i=1}^{N} tr({\varvec{Z}}^\top {\varvec{R}}_i^\top ({\varvec{X}}_i^\top \varvec{\Sigma }_n^{-1} {\varvec{M}} {\varvec{Z}} {\varvec{Z}}^\top \varvec{\Sigma }_m^{-1} {\varvec{Z}} + k {\varvec{F}})) \ne \sum _{i=1}^{N} tr( {\varvec{R}}_i^\top ({\varvec{X}}_i^\top \varvec{\Sigma }_n^{-1} {\varvec{M}} \varvec{\Sigma }_m^{-1} + k {\varvec{F}})) \end{aligned}$$

since the cyclic permutation invariance property of the trace does not work as Lemma 1 having the additional term $k tr( {\varvec{F}} {\varvec{R}}_i)$.

In addition, recalling Lemma 4, the solution for ${\varvec{R}}_i$ is unique if and only if ${\varvec{X}}_i^\top \varvec{\Sigma }_n^{-1} {\varvec{M}} \varvec{\Sigma }_m^{-1}+ k {\varvec{F}}$ has full rank. If ${\varvec{F}}$ is defined with full rank, so $\varvec{{\tilde{X}}}_i^\top \varvec{{\tilde{M}}} + k {\varvec{F}}$, and the solution for $R_i$ is unique. Furthermore, recalling Jupp and Mardia (1979), the mode of the von Mises–Fisher is the orientation part of location matrix parameter. Because the polar decomposition of ${\varvec{F}}^\star$ is unique, the maximum a posteriori estimate is unique. $\square$

To sum up, the ProMises model enables resolving the non-identifiability of $\varvec{R_i}$ that characterizes the perturbation model. The prior information inserted in the model permits guidance of the estimation process, computing a unique and interpretable data orthogonal transformation. Finally, all these properties are reached without complicating the estimation process of the perturbation model; we only modify the singular value decomposition step of Algorithm 1.

## Efficient ProMises Model

The framework depicted above can be applied both in low- and high-dimensional settings. However, the extension to the high-dimensional case does not come for free if the perturbation model is used. When $n<m$, rank equals *n*, the identifiability of the solution is lost even when the nuisance parameters are known. The lemma below formally states:

### Lemma 4

Consider ${\varvec{X}}_i \in \mathrm{I\!R}^{n \times m}$, if $n < m$, then the maximum likelihood estimate for ${\varvec{R}}_i$ defined in Theorem 1 is not unique.

Although the ProMises model provides unique solutions even in high-dimensional frameworks, a second issue remains prominent: the computational load. At each step, the presented algorithms perform *N* singular values decompositions of $m\times m$ matrices, which have a polynomial-time complexity $O(m^3)$. When *m* becomes large, as in fMRI data where *m* is a few hundred thousands, the computation runtime, and the required storing memory, becomes inadmissible.

This section proposes the Efficient ProMises model, which resolves the two above points. The method is efficient in terms of space and time complexity and fixes the non-identifiability of ${\varvec{R}}_i$. The algorithm allows a faster and more accessible shape analysis without loss of information in the case of $n \ll m$. It essentially merges the thin singular value decomposition (Bai et al., 2000) with the Procrustes problem.

In practice, the Efficient ProMises approach projects the matrices ${\varvec{X}}_i$ into an *n*-lower-dimensional space using a specific semi-orthogonal transformation (Abadir & Magnus, 2005; Groß et al., 1999) ${\varvec{Q}}_i$, with dimensions $m \times n$, which preserve all the data’s information. It aligns, then, the reduced $n\times n$ matrices $\{{\varvec{X}}_i {\varvec{Q}}_i \in \mathrm{I\!R}^{n \times n }\}_{i = 1, \dots , N}$ by the perturbation or ProMises model. Finally, it projects the aligned matrices back to the original $n\times m$-size matrices $\{{\varvec{X}}_i \in \mathrm{I\!R}^{n \times m }\}_{i = 1, \dots , N}$ using the transpose of $\{{\varvec{Q}}_i\}_{i = 1, \dots , N}$.

The following theorem proves that the maximum defined in Eq. (3) using $\{{\varvec{X}}_i {\varvec{Q}}_i \in \mathrm{I\!R}^{n \times n }\}_{i = 1, \dots , N}$ equals the original maximum because the Procrustes problem analyzes the first $n \times n$ dimensions of ${\varvec{R}}_i$. The maximum remains the same if we multiply $\{{\varvec{X}}_i \in \mathrm{I\!R}^{n \times m }\}_{i = 1, \dots , N}$ by ${\varvec{Q}}_i$.

### Theorem 3

Consider the perturbation model in Definition 2 with $\varvec{\Sigma }_m = \sigma ^2 {\varvec{I}}_m$ and the thin singular value decompositions of ${\varvec{X}}_i = {\varvec{L}}_i {\varvec{S}}_i {\varvec{Q}}_i^\top$ for each $i = 1, \dots , N$, where ${\varvec{Q}}_i$ has dimensions $m \times n$. The following holds

$$\begin{aligned} \max _{{\varvec{R}}_i \in {\mathcal {O}}(m)} tr({\varvec{R}}_i^\top {\varvec{X}}_i^\top \varvec{\Sigma }_n^{-1} {\varvec{X}}_j \varvec{\Sigma }_m^{-1}) = \max _{{\varvec{R}}_i^{\star } \in {\mathcal {O}}(n)} tr({\varvec{R}}_i^{ \star \top } {\varvec{Q}}_i^\top {\varvec{X}}_i^\top \varvec{\Sigma }_n^{-1} {\varvec{X}}_j \varvec{\Sigma }_m^{-1} {\varvec{Q}}_j^\top ). \end{aligned}$$

Additionally, the condition for the existence of $\hat{\varvec{\Sigma }}_n$ by Dutilleul (1999) is satisfied because ${\varvec{X}}_i$ now has dimensions $n \times n$, and the functional alignment needs at least $N = 2$ observations.

So, Theorem 3 is used to define an Efficient version of the ProMises model.

### Lemma 5

Consider the assumptions of Theorem 3, then

$$\begin{aligned} \max _{{\varvec{R}}_i \in {\mathcal {O}}(m)} tr({\varvec{R}}_i^\top {\varvec{X}}_i^\top \varvec{\Sigma _n}^{-1} {\varvec{X}}_j \varvec{\Sigma }_m^{-1} + k {\varvec{F}}) = \max _{{\varvec{R}}_i^{\star } \in {\mathcal {O}}(n)} tr\{{\varvec{R}}_i^{\star \top } ({\varvec{Q}}_i^\top {\varvec{X}}_i^\top \varvec{\Sigma }_n^{-1} {\varvec{X}}_j \varvec{\Sigma }_m^{-1} {\varvec{Q}}_j^\top + k {\varvec{F}}^\star )\}, \end{aligned}$$

where $${{F}} \in \mathrm {I\!R}^{m \times m}$$ and $${{F}}^\star = {{Q}}_i^\top {{F}} {{Q}}_j \in \mathrm {I\!R}^{n \times n}$$.

Proofs of Theorem 3 and Lemma 5 are shown in the supplementary material, whereas here, we make some further considerations about the proposed method.

At first glance, the assumption $\varvec{\Sigma }_m = \sigma ^2 {\varvec{I}}_m$ may dilute the resul’s impact. However, this assumption does not imply that the data are column-wise independent because this dependence is modeled by ${\varvec{R}}_i$. Additionally, the joint and accurate estimate of $\varvec{\Sigma }_m$, $\varvec{\Sigma }_n$ and ${\varvec{R}}_i$ requires a large number of observations. So, it is common in real applications to set $\varvec{\Sigma }_m$ and $\varvec{\Sigma }_n$ to be proportional to the identities in Procrustes-like problems (Haxby et al., 2020). When the model is high-dimensional, this problem becomes even more pronounced because of the huge number of parameters to be estimated.

The Efficient ProMises approach reaches the same maximum while working in the reduced space of the first *n* eigenvectors, which contains all the information, instead of the full data set. Therefore, the original problem estimates orthogonal matrices of size $m\times m$: ${\varvec{R}}_i \in {\mathcal {O}}(m)$, whereas the Efficient solution provides a set of orthogonal matrices of size $n\times n$: ${\varvec{R}}_i^{\star } \in {\mathcal {O}}(n)$. Even when the solution is projected back into the $m\times m$ space through ${\varvec{Q}}_i{\varvec{R}}_i^{\star }{\varvec{Q}}_i^\top$, the rank remains *n*, whereas the matrices of the original solutions have rank *m*. This should clarify that the Efficient approach reaches the same fit to the data under a different set of constraints that is $n\times n$ orthogonal matrices instead of $m\times m$ matrices; hence, the solutions of the two algorithms will not be identical.

Then, we add on Algorithm 1 the lines used to reduce the dimensions of ${\varvec{X}}_i$, and we modify Line 5 to insert the prior information:
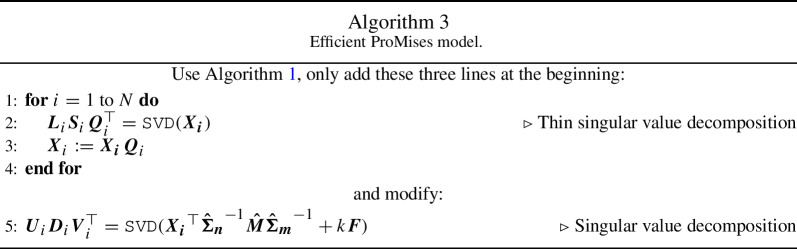


The Efficient approach reduces the time complexity from ${\mathcal {O}}(m^3)$ to ${\mathcal {O}}(m n^2)$ and the space complexity from ${\mathcal {O}}(m^2)$ to ${\mathcal {O}}(m n)$.

## Functional Magnetic Resonance Imaging Data Application

### Motivation

The alignment problem is recognized in fMRI multi-subject studies because the brain’s anatomical and functional structures vary across subjects. The most used anatomical alignments are the Talairach normalization (Talairach & Tournoux, 1988) and the Montréal Neurological Institute (MNI) space normalization (Jenkinson et al., 2002), where the brain images are aligned to an anatomical template by affine transformations using a set of major anatomical landmarks. However, this alignment does not explore the between-subjects variability in anatomical positions of the functional loci. The functional brain regions are not consistently placed on the anatomical landmarks defined by the Talairach and MNI templates. The anatomical alignment is then an approximate inter-subject registration of the functional cortical areas. Haxby et al. (2011) proved that functional brain anatomy exhibits a regular organization at a fine spatial scale shared across subjects.

Therefore, we can assume that anatomical and functional structures are subject-specific (Conroy et al., 2009; Sabuncu et al., 2010) and that the neural activities in different brains are noisy rotations of a common space (Haxby et al., 2011). Functional alignments (e.g., Procrustes methods) attempt to rotate the neural activities to maximize similarity across subjects.

Specifically, each subject’s brain activation can be represented by a matrix, where the rows represent the stimuli/time points, and the columns represent the voxels. The stimuli are time-synchronized among subjects, so we have correspondence among the matrices’ rows. However, the columns are not assumed to be in correspondence among subjects, as explained before. Each time series of brain activation (i.e., each of the matrices’ columns) represents the voxels’ functional characteristics that the anatomical normalization fails to align. We aim to represent the neural responses to stimuli into a common high-dimensional space, rather than in a canonical anatomical space that does not consider the variability of functional topographies loci.

**Fig. 2:**
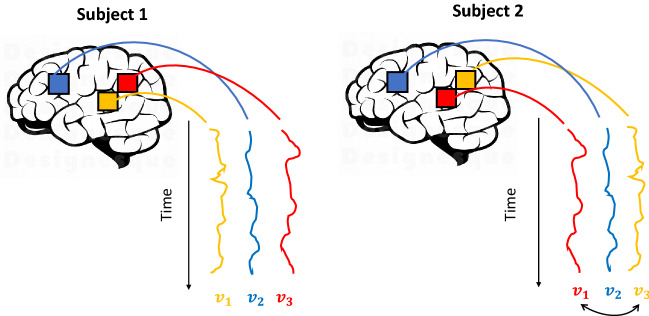
Illustration of functional misalignment between fMRI images, where three voxels’ time series are plotted considering two subjects. The time series of voxels $v_1$ and $v_3$ of the second subject are swapped with respect to the first subject.

Figure 2 shows three voxels’ neural activities (i.e., $v_1$, $v_2$, and $v_3$) three columns of the data matrix in two subjects recorded across time. The functional pattern of $v_2$ is equal across subjects, whereas $v_1$ and $v_3$ are swapped. A rotation matrix can resolve this misalignment, with the swap being a particular case of the rotation matrix. For further details about the motivation in using functional Procrustes-based alignment in fMRI studies, see Haxby et al. (2020).

### Data Description

We apply the proposed method to data from Pernet et al. (2015), available at https://openneuro.org/datasets/ds000158/versions/1.0.0. The study consists of neural activations of 218 subjects passively listening to vocal (i.e., speech) and nonvocal sounds. Because the application has had a mere illustrative purpose, we choose to use a small number of subjects (18) to facilitate the example’s reproducibility by the readers. We preprocessed the data using the Functional MRI of the Brain Software Library (FSL) (Jenkinson et al., 2012) using a standard processing procedure (i.e., high-pass filtering, brain extraction, spatially smoothing, registration to standard MNI space, dealing with motion and differences in slice acquisition time). Anatomical and functional alignment (based on the ProMises model) is compared, having images preprocessed in the same way, but in one case, the functional alignment is applied, while in the other case not. For details about the experimental design and data acquisition, please see Pernet et al. (2015).

### Functional Connectivity

We performed region of interest and seed-based correlation analysis (Cordes et al., 2000). The seed-based correlation map shows the level of functional connectivity between a seed and every voxel in the brain, whereas the region of interest analysis expresses the functional correlation between predefined regions of interest coming from a standard atlas. The analysis process is defined as follows: First, the subject images are aligned using Algorithm 3, then the element-wise arithmetic mean across subjects is calculated, and finally, the functional connectivity analysis is developed on this average matrix.

**Fig. 3:**
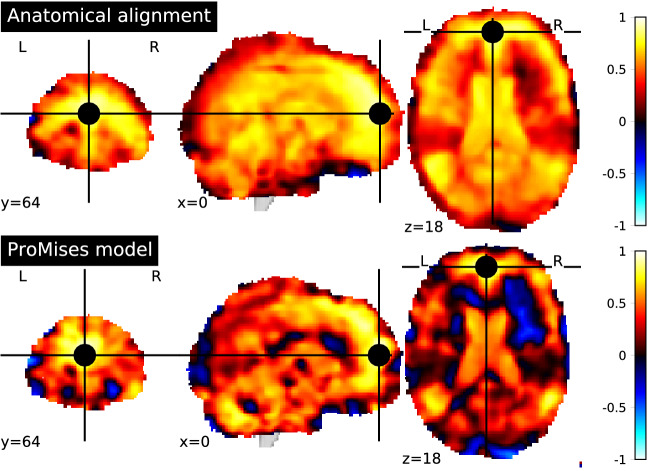
Seed-based correlation map for ${\varvec{M}}$, using data only aligned anatomically (top figure), and data also functionally aligned by the Efficient ProMises model (bottom figure). The black point refers to the seed used (i.e., frontal pole with MNI coordinates (0, 64, 18)). So, the brain map indicates the level of correlation between each voxel and the frontal pole.

We take the frontal pole as seed, being a region with functional diversity (Liu et al., 2013). The anatomical alignment considered here refers to the MNI space normalization (Jenkinson et al., 2002). Figure 3 shows the correlation values between the seed and each voxel in the brain using data without functional alignment (top of Fig. 3) and with functional alignment using the Efficient ProMises model (bottom of Fig. 3). The first evidence is that the functional alignment produces more interpretable maps, where the various regions, such as the superior temporal gyrus, are delineated by marked spatial edges, while the non-aligned map produces more spread regions, hence being less interpretable. It is interesting to evaluate the regions more correlated with the frontal pole, for example, the superior temporal gyrus. This region is associated with the processing of auditory stimuli. The correlation of the superior temporal gyrus with the seed is clear in the bottom part of Fig. 3, where functionally aligned images are used.

**Fig. 4:**
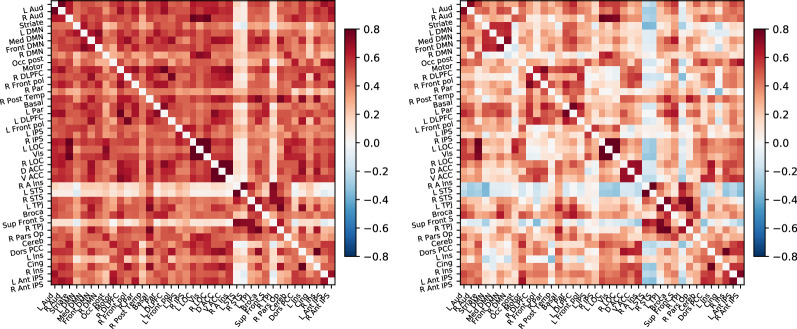
Correlation matrix for ${\varvec{M}}$, using data only aligned anatomically (left figure) and data also functionally aligned by the Efficient ProMises model (right figure). The cells of the matrix represent the correlation between the regions (represented by the row/column labels) of the Varoquaux et al. (2011)’s atlas.

In contrast, the region of interest correlations analysis shows the integration mechanisms between specialized brain areas. Figure 4 indicates the correlation matrices of time-series extracted from the 39 main regions of the atlas of Varoquaux et al. (2011). Using functionally aligned data (right side of Fig. 4), we can see delineated blocks of synchronized regions that can be interpreted as large-scale functional networks. Instead, using data without functional alignment (left side of Fig. 4) the distinctions between blocks are clearly worse. Using functionally aligned data, the left and right visual systems, composed of the dorsolateral prefrontal cortex (DLPFC), frontal pole (Front pol), and parietal (Par), are clearly visible, whereas in the analysis using functionally nonaligned data, this distinction is hidden by noise.

The preprocessed data are available on the GitHub repository: http://github.com/angeella/fMRIdata, as well as the code used to perform functional connectivity: http://github.com/angeella/ProMisesModel/Code/Auditory.

## Discussion

The ProMises model provides a methodologically grounded approach to the Procrustes problem allowing functional alignment on high-dimensional data in a computationally efficient way. The issues of the perturbation model (Goodall, 1991)—non-uniqueness, critical interpretation, and inapplicability when $n \ll m$—are completely surpassed thanks to our Bayesian extension. Indeed, the ProMises method returns unique and interpretable orthogonal transformations, and its efficient approach extends the applicability to high-dimensional data. The presented method is particularly useful in fMRI data analysis because it allows the functional alignment of images having roughly $200 \times$ 200,000 dimensions, obtaining a unique representation of the aligned images in the brain space and a unique interpretation of the related results.

In the application example presented in Sect. 4, a subsample was analyzed. However, the algorithm has a linear growth in *N*, and therefore, it is not a problem to work with larger samples. Also, the algorithm permits a parallel computation for the subjects.

The Bayesian framework gives the user the advantage and the duty to insert prior information into the model through *k* and ${\varvec{F}}$. The parameter *k* plays the role of regularization parameter, which is rarely known a priori. We estimated it by cross-validation, although it may be interesting to adapt approximations-based methods (e.g., generalized cross-validation) to reduce the computational burden. Alternatively, we could assume a prior distribution taking values in ${\mathbb {R}}^{+}$ for the regularization parameter *k* and proceed to jointly estimate this parameter as well. More interestingly, the matrix ${\varvec{F}}$ addresses the estimate of the optimal rotations, which is favorable in the analysis of fMRI data because, in this context, the variables have a spatial anatomical location. In the example in Sect. 4, our definition of ${\varvec{F}}$ favors the combination of voxels with equal location. However, a more thoughtful specification can entirely exploit the voxels’ specific spatial position in the anatomical template. This opens up the possibility to explore various specifications of ${\varvec{F}}$ and will be the subject of further research.

## Supplementary Information

Below is the link to the electronic supplementary material.

**Supplementary Materials:** Supplementary material includes the proofs of theorems and lemmas. (pdf 232KB)
