# Supplementary material for: Procrustes Analysis for High-Dimensional Data
Source: Psychometrika. 2022 May 18;87(4):1422–38. doi: 10.1007/s11336-022-09859-5 (PMC9636303; doi:10.1007/s11336-022-09859-5)
Supplement: Supplementary file 1 — Supplementary Materials: Supplementary material includes the proofs of theorems and lemmas. (pdf 232KB) [file 11336_2022_9859_MOESM1_ESM.pdf]

SUPPLEMENTAL MATERIAL FOR "PROCRUSTES ANALYSIS FOR HIGH-DIMENSIONAL DATA"

A. Proof of Theorems and Lemmas

Theorem 1. (Theobald & Wuttke (2006)) Consider the perturbation model described in Definition 2, and the singular value decomposition $\boldsymbol{X}_i^\top \boldsymbol{\Sigma}_n^{-1} \boldsymbol{M} \boldsymbol{\Sigma}_m^{-1} = \boldsymbol{U}_i \boldsymbol{D}_i \boldsymbol{V}_i^\top$. So, the maximum likelihood estimators equal $\hat{\boldsymbol{R}}_i = \boldsymbol{U}_i \boldsymbol{V}_i^\top$, and $\hat{\alpha}_{i_{\hat{\boldsymbol{R}}_i}} = ||\Sigma_m^{-1/2} \hat{\boldsymbol{R}}_i^\top \boldsymbol{X}_i^\top \boldsymbol{\Sigma}_{\boldsymbol{n}}{}^{-1/2}||^2 / tr(\boldsymbol{D}_i)$.

Proof. The proof comes directly from Theobald & Wuttke (2006), we report here the final part that we will use for other proofs. We can write Equation (1) as:

$$\frac{1}{\alpha_i} \boldsymbol{X}_i \boldsymbol{R}_i - \boldsymbol{M} = \boldsymbol{E}_i \sim \mathcal{MN}(0, \boldsymbol{\Sigma}_n, \boldsymbol{\Sigma}_m).$$

The log-likelihood for $\boldsymbol{R}_i$ equals:

$$\ell(\boldsymbol{R}_i) = -\frac{1}{2} \sum_{i=1}^{N} tr\{\boldsymbol{\Sigma}_m^{-1} (\frac{1}{\alpha_i} \boldsymbol{X}_i \boldsymbol{R}_i - \boldsymbol{M})^\top \boldsymbol{\Sigma}_n^{-1} (\frac{1}{\alpha_i} \boldsymbol{X}_i \boldsymbol{R}_i - \boldsymbol{M})\} + C,$$

where $(\boldsymbol{M}, \boldsymbol{\Sigma}_n, \boldsymbol{\Sigma}_m)$ are known nuisance parameter and C is a constant value. So, we have

$$\ell(\boldsymbol{R}_i) = \frac{1}{\alpha_i} tr\{\boldsymbol{R}_i^\top \boldsymbol{X}_i^\top \boldsymbol{\Sigma}_n^{-1} \boldsymbol{M} \boldsymbol{\Sigma}_m^{-1}\} + C^\star,$$

where $C^\star$ is a constant. The maximization of the log-likelihood function $\ell(\boldsymbol{R}_i)$ leads to

$$\begin{aligned}
\hat{\boldsymbol{R}}_i &= \arg \max_{\boldsymbol{R}_i \in \mathcal{O}(m)} < \boldsymbol{R}_i, \boldsymbol{X}_i^\top \boldsymbol{\Sigma}_n^{-1} \boldsymbol{M} \boldsymbol{\Sigma}_m^{-1} > \\
&= \arg \max_{\boldsymbol{R}_i \in \mathcal{O}(m)} < \boldsymbol{R}_i, \boldsymbol{U}_i \boldsymbol{D}_i \boldsymbol{V}_i >= \arg \max_{\boldsymbol{R}_i \in \mathcal{O}(m)} < \boldsymbol{D}_i, \boldsymbol{U}_i^\top \boldsymbol{R}_i \boldsymbol{V}_i > \\
&= \arg \max_{\boldsymbol{R}_i \in \mathcal{O}(m)} < \boldsymbol{D}_i, \boldsymbol{R}_i^o >= \boldsymbol{U}_i \boldsymbol{V}_i^\top, \qquad (1)
\end{aligned}$$

where $\boldsymbol{R}_i^o = \boldsymbol{U}_i^\top \boldsymbol{R}_i \boldsymbol{V}_i \in \mathcal{O}(m)$. The step (1) is proved by Gower & Dijksterhuis (2004), i.e., $< \boldsymbol{D}_i, \boldsymbol{R}_i^o >$ is maximum when $\boldsymbol{R}_i^o = \boldsymbol{I}_m$, giving $\boldsymbol{I}_m = \boldsymbol{U}_i^\top \hat{\boldsymbol{R}}_i \boldsymbol{V}_i$. We can note that the maximum likelihood estimator $\hat{\boldsymbol{R}}_i$ does not depend on α_i.

Consider the profile log-likelihood for α_i:

$$\begin{aligned}
\ell_p(\alpha_i) &= -\frac{1}{2}\sum_{i=1}^N tr\{\boldsymbol{\Sigma}_m^{-1/2}(\frac{1}{\alpha_i}\boldsymbol{X}_i\hat{\boldsymbol{R}}_i - \boldsymbol{M})^\top \boldsymbol{\Sigma}_n^{-1/2}\boldsymbol{\Sigma}_n^{-1/2}(\frac{1}{\alpha_i}\boldsymbol{X}_i\hat{\boldsymbol{R}}_i - \boldsymbol{M})\boldsymbol{\Sigma}_m^{-1/2}\} + S \\
&= -\frac{1}{2}\{\sum_{i=1}^N \frac{1}{\alpha_i^2} tr(\boldsymbol{\Sigma}_m^{-1/2}\hat{\boldsymbol{R}}_i^\top \boldsymbol{X}_i^\top \boldsymbol{\Sigma_n}^{-1/2}\boldsymbol{\Sigma}_n^{-1/2}\boldsymbol{X}_i\hat{\boldsymbol{R}}_i\boldsymbol{\Sigma}_m^{-1/2}) \\
&\quad + tr(\boldsymbol{\Sigma}_m^{-1/2}\boldsymbol{M}^\top \boldsymbol{\Sigma}_n^{-1/2}\boldsymbol{\Sigma}_n^{-1/2}\boldsymbol{M}\boldsymbol{\Sigma}_m^{-1/2}) \\
&\quad - 2\frac{1}{\alpha_i} tr(\boldsymbol{\Sigma}_m^{-1/2}\hat{\boldsymbol{R}}_i^\top \boldsymbol{X}_i^\top \boldsymbol{\Sigma}_n^{-1/2}\boldsymbol{\Sigma}_n^{-1/2}\boldsymbol{M}\boldsymbol{\Sigma}_m^{-1/2})\} + S \\
&= \sum_{i=1}^N -\frac{1}{2\alpha_i^2}||\boldsymbol{\Sigma}_m^{-1/2}\hat{\boldsymbol{R}}_i^\top \boldsymbol{X}_i^\top \boldsymbol{\Sigma}_n^{-1/2}||^2 + \frac{1}{\alpha_i} tr(\boldsymbol{D}_i) + S^\star,
\end{aligned}$$

where S, and $S^\star$ are constant values. Taking the first derivative, we have:

$$\begin{aligned}
\frac{\partial \ell_p(\alpha_i)}{\partial \alpha_i} &= \alpha_i^{-1}||\boldsymbol{\Sigma}_m^{-1/2}\hat{\boldsymbol{R}}_i^\top \boldsymbol{X}_i^\top \boldsymbol{\Sigma}_n^{-1/2}||^2 - tr(\boldsymbol{D}_i) \\
\hat{\alpha_i}_{\hat{R}_i} &= \frac{||\boldsymbol{\Sigma}_m^{-1/2}\hat{\boldsymbol{R}}_i^\top \boldsymbol{X}_i^\top \boldsymbol{\Sigma_n}^{-1/2}||^2}{tr(\boldsymbol{D}_i)},
\end{aligned}$$

having $\hat{\boldsymbol{R}}_i = \boldsymbol{U}_i\boldsymbol{V}_i^\top$ and $\alpha_i \in \mathbb{R}^+$.

Lemma 2. Consider the perturbation model of Definition 2, with $\boldsymbol{R}_i$ distributed accordingly to (4), then the posterior distribution $f(\boldsymbol{R}_i|k, \boldsymbol{F}, \boldsymbol{X}_i)$ is conjugate distribution to the von Mises-Fisher prior distribution with location posterior parameter equalling the following:

$$\boldsymbol{F}^\star = \boldsymbol{X_i}^\top \boldsymbol{\Sigma}_n^{-1}\boldsymbol{M}\boldsymbol{\Sigma}_m^{-1} + k\boldsymbol{F}.$$

Proof. The joint posterior distribution is defined as

$$\prod_{i=1}^{N} f(\boldsymbol{R}_i|\boldsymbol{X}_i, \boldsymbol{M}, \boldsymbol{\Sigma}_m, \boldsymbol{\Sigma}_n, k, \boldsymbol{F}) = \prod_{i=1}^{N} \exp[-\frac{1}{2} tr\{\boldsymbol{\Sigma}_m^{-1/2}(\frac{1}{\alpha_i}\boldsymbol{X}_i\boldsymbol{R}_i - \boldsymbol{M})^\top \boldsymbol{\Sigma}_n^{-1/2}\boldsymbol{\Sigma}_n^{-1/2}$$
$$(\frac{1}{\alpha_i}\boldsymbol{X_i}\boldsymbol{R_i} - \boldsymbol{M})\boldsymbol{\Sigma}_m^{-1/2}\}]$$
$$\cdot \exp\left\{k tr(\boldsymbol{F}^\top \boldsymbol{R}_i)\right\} \cdot C$$
$$= \exp(-\sum_{i=1}^{N} \frac{1}{2}\Psi_i)$$
$$\cdot \exp(\sum_{i=1}^{N} < \boldsymbol{X}_i^\top \boldsymbol{\Sigma}_n^{-1} \boldsymbol{M} \boldsymbol{\Sigma}_m^{-1} + k\boldsymbol{F}, \boldsymbol{R}_i >), \tag{2}$$

where $\Psi_i = f(X_i)$ and C is a constant value. The quantity (2) is a kernel of a matrix von Mises-Fisher distribution with location parameter equals

$$\boldsymbol{F}^\star = \boldsymbol{X}_i^\top \boldsymbol{\Sigma}_n^{-1} \boldsymbol{M} \boldsymbol{\Sigma}_m^{-1} + k\boldsymbol{F}.$$

Theorem 2. The ProMises model is defined as the perturbation model specified in Definition 2 imposing the prior distribution (8) for $\alpha_i \boldsymbol{R}_i$. Let the singular value decomposition of $\boldsymbol{X}_i^\top \boldsymbol{\Sigma}_n^{-1} \boldsymbol{M} \boldsymbol{\Sigma}_m^{-1} + k\boldsymbol{F}$ be $\boldsymbol{U}_i \boldsymbol{D}_i \boldsymbol{V}_i^\top$. Then, the maximum a posteriori estimators equal $\hat{\boldsymbol{R}}'_i = \boldsymbol{U}_i \boldsymbol{V}_i^\top$, and $\hat{\alpha}_{i}{}'_{\hat{\boldsymbol{R}}'_i} = ||\boldsymbol{\Sigma}_m^{-1/2} \hat{\boldsymbol{R}}'_i \boldsymbol{X}_i^\top \boldsymbol{\Sigma}_n^{-1/2}||^2 / tr(\boldsymbol{D}_i)$.

Proof. Consider the same assumption of Theorem 1, the log-posterior distribution for $\boldsymbol{R}_i$ and α_i equals:

$$\log f(\alpha_i, \boldsymbol{R_i}|\boldsymbol{X_i}, \boldsymbol{M}, \boldsymbol{\Sigma}_m, \boldsymbol{\Sigma}_n, k, \boldsymbol{F}) = -\frac{1}{2}\sum_{i=1}^{N} tr\{\boldsymbol{\Sigma}_m^{-1}(\frac{1}{\alpha_i}\boldsymbol{X}_i\boldsymbol{R}_i - \boldsymbol{M})^\top \boldsymbol{\Sigma}_n^{-1}(\frac{1}{\alpha_i}\boldsymbol{X}_i\boldsymbol{R}_i - \boldsymbol{M})$$
$$- 2\frac{k}{\alpha_i}\boldsymbol{F}^\top \boldsymbol{R}_i\} - \log(\alpha_i) + K,$$

where K is a constant value. Following the same steps of Theorem 1's proof, the maximum a

posteriori estimate equals

$$
\begin{aligned}
\hat{\boldsymbol{R}}'_i &= \arg\max_{\boldsymbol{R}_i \in \mathcal{O}(m)} < \boldsymbol{R}_i, \boldsymbol{X}_i^\top \boldsymbol{\Sigma}_n^{-1} \boldsymbol{M} \boldsymbol{\Sigma}_m^{-1} > +k < \boldsymbol{R}_i, \boldsymbol{F} > \\
&= \arg\max_{\boldsymbol{R}_i \in \mathcal{O}(m)} < \boldsymbol{R}_i, \boldsymbol{X}_i^\top \boldsymbol{\Sigma}_n^{-1} \boldsymbol{M} \boldsymbol{\Sigma}_m^{-1} + k\boldsymbol{F} > \\
&= \arg\max_{\boldsymbol{R}_i \in \mathcal{O}(m)} < \boldsymbol{R}_i, \boldsymbol{U}_i \boldsymbol{D}_i \boldsymbol{V}_i >= \arg\max_{\boldsymbol{R}_i \in \mathcal{O}(m)} < \boldsymbol{D}_i, \boldsymbol{U}_i^\top \boldsymbol{R}_i \boldsymbol{V}_i > \\
&= \max_{\boldsymbol{R}_i \in \mathcal{O}(m)} < \boldsymbol{D}_i, \boldsymbol{R}_i^o >= \boldsymbol{U}_i \boldsymbol{V}_i^\top, \qquad (3)
\end{aligned}
$$

where step (3) is proved in the same way as step (1) of Theorem 1' proof.

Then we compute the maximum a posteriori estimate for α_i:

$$
\begin{aligned}
\hat{\alpha}_{i_{\hat{\boldsymbol{R}}'_i}}' &= \arg\max_{\alpha_i \in \mathbb{R}^+} -\frac{1}{2\alpha_i^2} ||\boldsymbol{\Sigma}_m^{-1/2} \hat{\boldsymbol{R}}'^\top{}_i \boldsymbol{X}_i^\top \boldsymbol{\Sigma}_{\boldsymbol{n}}{}^{-1/2}||^2 + \frac{1}{\alpha_i} < \hat{\boldsymbol{R}}'^\top{}_i, \boldsymbol{X}_i^\top \boldsymbol{\Sigma}_n^{-1} \boldsymbol{M} \boldsymbol{\Sigma}_m^{-1} > \\
&+ \frac{k}{\alpha_i} tr(\boldsymbol{F}^\top \hat{\boldsymbol{R}}'_i) - \log(\alpha_i) + P,
\end{aligned}
$$

where P is a constant value. Compute the first derivative and set it to zero:

$$
\alpha_i^{-2} ||\boldsymbol{\Sigma}_m^{-1/2} \hat{\boldsymbol{R}}'^\top{}_i \boldsymbol{X}_i^\top \boldsymbol{\Sigma}_n^{-1/2}||^2 - \alpha_i^{-1} < \hat{\boldsymbol{R}}'_i, \boldsymbol{X}_i^\top \boldsymbol{\Sigma}_n^{-1} \boldsymbol{M} \boldsymbol{\Sigma}_m^{-1} + k\boldsymbol{F} > -1 = 0,
$$

so, applying the Viéte theorem (Viéte, 1646), $\hat{\alpha}_{i_{\hat{\boldsymbol{R}}'_i}}$ equals:

$$
\hat{\alpha}_{i_{\hat{\boldsymbol{R}}'_i}} = \frac{||\boldsymbol{\Sigma}_m^{-1/2} \hat{\boldsymbol{R}}'^\top{}_i \boldsymbol{X}_i^\top \boldsymbol{\Sigma}_n^{-1/2}||^2}{tr(\boldsymbol{D}_i)},
$$

under the condition $\dfrac{tr(\boldsymbol{D}_i)}{||\Sigma_m^{-1/2} \hat{\boldsymbol{R}}'^\top{}_i \boldsymbol{X}_i^\top \boldsymbol{\Sigma}_n^{-1/2}||^2} \gg \dfrac{1}{tr(\boldsymbol{D}_i)}$, and $\boldsymbol{D}_i$ coming from the singular value decomposition of $\boldsymbol{X}_i^\top \boldsymbol{\Sigma}_n^{-1} \boldsymbol{M} \boldsymbol{\Sigma}_m^{-1} + k\boldsymbol{F}$.

Lemma 4. Consider $\boldsymbol{X}_i \in \mathbb{R}^{n \times m}$, if $n < m$, then the maximum likelihood estimate for $\boldsymbol{R}_i$ defined in Theorem 1 is not unique.

Proof. In practice, without loss of generality, the Procrustes problem can be resumed as:

$$
\max_{\boldsymbol{R}_i \in \mathcal{O}(m)} tr(\boldsymbol{A}_i^\top \boldsymbol{R}_i), \qquad (4)
$$

where $\boldsymbol{A}_i = \boldsymbol{X}_i^\top \boldsymbol{\Sigma}_n^{-1} \boldsymbol{M} \boldsymbol{\Sigma}_m^{-1}$. Trendafilov & Lippert (2002) and Myronenko & Song (2009, Lemma 1) proved that the solution for (4) is unique if and only if the matrix $\boldsymbol{A}_i$ has full rank. In Theorem 1 with $n < m$, $\boldsymbol{A}_i$ is equal to $\boldsymbol{X}_i^\top \boldsymbol{\Sigma}_n^{-1} \boldsymbol{M} \boldsymbol{\Sigma}_m^{-1}$ having rank lower than m, so the solution is not unique. Please refer to Trendafilov & Lippert (2002) and Myronenko & Song (2009, Lemma 1) for further details about the complete proof.

Theorem 3. Consider the perturbation model in Definition 2 with $\boldsymbol{\Sigma}_m = \sigma^2 \boldsymbol{I}_m$, and the thin singular value decompositions of $\boldsymbol{X}_i = \boldsymbol{L}_i \boldsymbol{S}_i \boldsymbol{Q}_i^\top$ for each $i = 1, \dots, N$, where $\boldsymbol{Q}_i$ has dimensions $n \times m$. The following holds

$$\max_{\boldsymbol{R}_i \in \mathcal{O}(m)} tr(\boldsymbol{R}_i^\top \boldsymbol{X}_i^\top \boldsymbol{\Sigma}_n^{-1} \boldsymbol{X}_j \boldsymbol{\Sigma}_m^{-1}) = \max_{\boldsymbol{R}_i^\star \in \mathcal{O}(n)} tr(\boldsymbol{R}_i^{\star\top} \boldsymbol{Q}_i^\top \boldsymbol{X}_i^\top \boldsymbol{\Sigma}_n^{-1} \boldsymbol{X}_j \boldsymbol{\Sigma}_m^{-1} \boldsymbol{Q}_j^\top).$$

Proof. Without loss of generality we consider $\boldsymbol{\Sigma}_m = \sigma^2 \boldsymbol{I}_m$, and so the following objective function to maximize

$$tr(\boldsymbol{R}_i^\top \boldsymbol{X}_i^\top \boldsymbol{\Sigma}_n^{-1} \boldsymbol{X}_j \sigma^2 \boldsymbol{I}_m).$$

We note that it is equivalent to maximize

$$tr(\boldsymbol{\Sigma}_n^{-1} \boldsymbol{X}_i \boldsymbol{R}_i \boldsymbol{X}_j^\top) \tag{5}$$

thanks to the trace's proprieties.

Let consider the full singular value decomposition $\boldsymbol{X}_i = \boldsymbol{L}_i \boldsymbol{S}_i \boldsymbol{C}_i^\top$, where $\boldsymbol{S}_i \in \mathbb{R}^{n \times m}$. The $\boldsymbol{S}_i$ matrix is defined as

$$\boldsymbol{S}_i = [\boldsymbol{S}_i^\star \ \boldsymbol{O}],$$

where $\boldsymbol{S}_i^\star \in \mathbb{R}^{n \times n}$ and $\boldsymbol{O}$ is a matrix of zero with $n \times (m - n)$ dimensions, since $rank(\boldsymbol{X}_i) = n \ \forall i = 1, \dots, N$. Therefore, Expression (5) equals

$$tr(\boldsymbol{\Sigma}_n^{-1} \boldsymbol{X}_i \boldsymbol{R}_i \boldsymbol{X}_j^\top) = tr(\boldsymbol{\Sigma}_n^{-1} \boldsymbol{L}_i \boldsymbol{S}_i \boldsymbol{C}_i^\top \boldsymbol{R}_i \boldsymbol{C}_j \boldsymbol{S}_j^\top \boldsymbol{L}_j^\top) = tr(\boldsymbol{\Sigma}_n^{-1} \boldsymbol{L}_i \boldsymbol{S}_i \boldsymbol{R}_i^o \boldsymbol{S}_j^\top \boldsymbol{L}_j^\top),$$

where $\boldsymbol{R}_i^o = \boldsymbol{C}_i^\top \boldsymbol{R}_i \boldsymbol{C}_j \in \mathcal{O}(m)$ being a product of orthogonal matrices.

Partitioning $\boldsymbol{R}_i^o$ in blocks, i.e.,

$$\begin{bmatrix} \boldsymbol{R}_{11i}^o \boldsymbol{R}_{12i}^o \\ \boldsymbol{R}_{21i}^o \boldsymbol{R}_{22i}^o \end{bmatrix} \tag{6}$$

where $\boldsymbol{R}_{11i}^o \in \mathbb{R}^{n\times n}$, $\boldsymbol{R}_{12i}^o \in \mathbb{R}^{n\times m-n}$, $\boldsymbol{R}_{21i}^o \in \mathbb{R}^{m-n\times n}$, and $\boldsymbol{R}_{22i}^o \in \mathbb{R}^{m-n\times m-n}$, we have:

$$\begin{aligned} \boldsymbol{\Sigma}_n^{-1}\boldsymbol{L}_i\boldsymbol{S}_i\boldsymbol{R}_i^o\boldsymbol{S}_j^\top\boldsymbol{L}_j^\top &= \boldsymbol{\Sigma}_n^{-1}\boldsymbol{L}_i[\boldsymbol{S}_i^\star\ \boldsymbol{O}]\begin{bmatrix} \boldsymbol{R}_{11}^o \boldsymbol{R}_{12}^o \\ \boldsymbol{R}_{21}^o \boldsymbol{R}_{22}^o \end{bmatrix}\begin{bmatrix} \boldsymbol{S}_j^{\top\star} \\ \boldsymbol{O}^\top \end{bmatrix}\boldsymbol{L}_j^\top \\ &= [\boldsymbol{\Sigma}_n^{-1}\boldsymbol{L}_i\boldsymbol{S}_i^\star\ \boldsymbol{O}]\begin{bmatrix} \boldsymbol{R}_{11i}^o \boldsymbol{R}_{12i}^o \\ \boldsymbol{R}_{21i}^o \boldsymbol{R}_{22i}^o \end{bmatrix}\begin{bmatrix} \boldsymbol{S}_j^{\top\star}\boldsymbol{L}_j^\top \\ \boldsymbol{O}^\top \end{bmatrix} \\ &= \boldsymbol{\Sigma}_n^{-1}\boldsymbol{L}_i\boldsymbol{S}_i^\star\boldsymbol{R}_{11i}^o\boldsymbol{S}_j^{\top\star}\boldsymbol{L}_j^\top. \end{aligned} \tag{7}$$

Then, we have

$$\begin{aligned} \max_{\boldsymbol{R}_i\in\mathcal{O}(m)} tr(\boldsymbol{\Sigma}_n^{-1}\boldsymbol{X}_i\boldsymbol{R}_i\boldsymbol{X}_j^\top) &= \max_{\boldsymbol{R}_{11i}^o\in\mathcal{O}(n)} tr(\boldsymbol{\Sigma}_n^{-1}\boldsymbol{L}_i\boldsymbol{S}_i^\star\boldsymbol{R}_{11i}^o\boldsymbol{S}_j^{\star\top}\boldsymbol{L}_j^\top) \\ &= \max_{\boldsymbol{R}_i^\star\in\mathcal{O}(n)} tr(\boldsymbol{\Sigma}_n^{-1}\boldsymbol{X}_i\boldsymbol{Q}_i\boldsymbol{R}_i^\star\boldsymbol{Q}_j^\top\boldsymbol{X}_j^\top). \end{aligned}$$

The last equality is due to

$$\boldsymbol{\Sigma}_n^{-1}\boldsymbol{X}_i\boldsymbol{Q}_i\boldsymbol{R}_i^\star\boldsymbol{Q}_j^\top\boldsymbol{X}_j^\top = \boldsymbol{\Sigma}_n^{-1}\boldsymbol{L}_i\boldsymbol{S}_i^\star\boldsymbol{Q}_i^\top\boldsymbol{Q}_i\boldsymbol{R}_i^\star\boldsymbol{Q}_j^\top\boldsymbol{Q}_j\boldsymbol{S}_j^\star\boldsymbol{L}_j^\top = \boldsymbol{\Sigma}_n^{-1}\boldsymbol{L}_i\boldsymbol{S}_i^\star\boldsymbol{R}_i^\star\boldsymbol{S}_j^{\star\top}\boldsymbol{L}_j^\top.$$

So essentially, only the first n dimensions are used in maximizing (5), if $n < m$ in all Procrustes-based problem.

Lemma 5. Consider the assumptions of Theorem 3, then

$$\max_{\boldsymbol{R}_i\in\mathcal{O}(m)} tr(\boldsymbol{R}_i^\top\boldsymbol{X}_i^\top\boldsymbol{\Sigma_n}^{-1}\boldsymbol{X}_j\boldsymbol{\Sigma}_m^{-1} + k\boldsymbol{F}) = \max_{\boldsymbol{R}_i^\star\in\mathcal{O}(n)} tr\{\boldsymbol{R}_i^{\star\top}(\boldsymbol{Q}_i^\top\boldsymbol{X}_i^\top\boldsymbol{\Sigma}_n^{-1}\boldsymbol{X}_j\boldsymbol{\Sigma}_m^{-1}\boldsymbol{Q}_j^\top + k\boldsymbol{F}^\star)\},$$

where $\boldsymbol{F} \in \mathbb{R}^{m\times m}$ and $\boldsymbol{F}^\star \in \mathbb{R}^{n\times n}$.

Proof. Without loss of generality we consider $\boldsymbol{\Sigma}_m = \sigma^2\boldsymbol{I}_m$, and $\boldsymbol{F}^\star = \boldsymbol{Q}_i^\top\boldsymbol{F}\boldsymbol{Q}_j$. So, the following objective function to maximize

$$\max_{\boldsymbol{R}_i^{\star} \in \mathcal{O}(n)} tr(\boldsymbol{R}_i^{\top\star} \boldsymbol{Q}_i^{\top} \boldsymbol{X}_i^{\top} \boldsymbol{\Sigma}_n^{-1} \boldsymbol{X}_j \boldsymbol{Q}_j) + ktr(\boldsymbol{R}_i^{\top\star} \boldsymbol{Q}_i^{\top} \boldsymbol{F} \boldsymbol{Q}_j). \tag{8}$$

The left part of the maximization (8) equals (7), while $ktr(\boldsymbol{R}_i^{\top\star} \boldsymbol{Q}_i^{\top} \boldsymbol{F} \boldsymbol{Q}_j)$ in $\mathcal{O}(n)$ is equivalent to $ktr(\boldsymbol{R}_i^{\top} \boldsymbol{F})$ in $\mathcal{O}(m)$.

References

Gower, J. C. & Dijksterhuis, G. B. (2004). *Procrustes problems*, Volume 30. Oxford University Press on Demand.

Jupp, P. E. & Mardia, K. V. (1979). Maximum likelihood estimators for the matrix von mises-fisher and bingham distributions. *The Annals of Statistics* **7**(3), 599–606.

Myronenko, A. & Song, X. (2009). On the closed-form solution of the rotation matrix arising in computer vision problems. *arXiv preprint arXiv:0904.1613* .

Theobald, D. L. & Wuttke, D. S. (2006). Empirical bayes hierarchical models for regularizing maximum likelihood estimation in the matrix gaussian procrustes problem. *Proceedings of the National Academy of Sciences* **103**(49), 18521–18527.

Trendafilov, N. T. & Lippert, R. A. (2002). The multimode procrustes problem. *Linear algebra and its applications* **349**(1–3), 245–264.

Viéte, F. (1646). *Opera mathematica.* F. van Schouten.
